# Combination of immune checkpoint blockade and targeted gene regulation of angiogenesis for facilitating antitumor immunotherapy

**DOI:** 10.3389/fbioe.2023.1065773

**Published:** 2023-03-13

**Authors:** Jing Zhan, Manli Zhang, Lili Zhou, Chuan He

**Affiliations:** ^1^ Department of Gastroenterology, The First Hospital of Jilin University, Changchun, China; ^2^ Department of Hepatology and Gastroenterology, The First Hospital of Jilin University, Changchun, China; ^3^ Department of Radiology, The First Hospital of Jilin University, Changchun, China

**Keywords:** *in situ* tumor vaccines, anti-angiogenesis, gene therapy, immunotherapy, immune checkpoint blockade

## Abstract

The rapid development of tumor immunotherapy has improved the management of patients with cancer. However, several key problems of tumor immunotherapy, including the insufficient activation of effector T cells, poor tumor invasion, and poor immune killing ability, lead to a low response rate. In the present study, a synergistic strategy was developed by combining *in situ* tumor vaccines, gene-mediated downregulation of tumor angiogenesis, and anti-PD-L1 therapy. *In situ* tumor vaccines and antitumor angiogenesis were achieved by codelivering unmethylated cytosine-phosphate-guanine (CpG) and vascular endothelial growth factor (VEGF)-silencing gene (shVEGF) *via* a hyaluronic acid (HA)-modified HA/PEI/shVEGF/CpG system. Necrotic tumor cells and CpG adjuvants formed *in situ* tumor vaccines and activated the host immune response. Moreover, VEGF silencing reduced tumor angiogenesis and prompted the homogeneous distribution of tumor blood vessels to facilitate immune cell infiltration. Meanwhile, anti-angiogenesis also improved the immunosuppressive tumor microenvironment. To further improve the specific tumor-killing effect, an anti-PD-L1 antibody was introduced for immune checkpoint blockade, thereby boosting antitumor immune responses. The combination therapy strategy presented in the present study could act in the multiple stages of the tumor immunotherapy cycle, which is expected to offer a new avenue for clinical tumor immunotherapy.

## 1 Introduction

Tumor immunotherapy is capable of eliminating tumor cells by restarting and maintaining normal antitumor immune responses. This method adds to the cancer treatment options of surgery, chemotherapy, and radiotherapy (Gong et al., 2021; Varade et al., 2021; Zaborowski et al., 2021). The entire tumor immune response can be divided into the following steps: necrotic tumor cells release tumor antigens; these tumor antigens are endocytosed by antigen-presenting cells (APCs), which are then processed and presented on the cell surface; APCs migrate to tumor-draining lymph nodes (LNs) and present tumor antigens to T cells, thereby activating T cells; activated T cells travel to the tumor area *via* the bloodstream; T cells infiltrate into the tumor and recognize tumor cells by forming an immune synapse; and T cells release perforin and granuloenzymes, which ultimately eliminate tumor cells (Xie et al., 2022). The killed tumor cells release more tumor antigens, and the immune circulation begins again until all the tumor cells are removed. The process of immunotherapy is complex and comprehensively regulated by various immune cells, cytokines, and the immune microenvironment. The most critical processes are the activation of effector T cells, infiltration of the tumor tissue, and activation of immune-mediated tumor killing (Wang et al., 2021).

There are many strategies for effector T-cell activation, including tumor vaccines (Chen et al., 2022); cytokine therapy (Hotz et al., 2021); chimeric antigen receptor T-Cell therapy (Car-T therapy) (Dhakal et al., 2021); and immunogenic cell death (ICD) induced by radiotherapy (Li et al., 2021), chemotherapy (Wu et al., 2020), phototherapy (Ni et al., 2021) or others (Kroemer et al., 2013). Tumor vaccines can activate the immune system to produce a specific response against tumor cells (Hu et al., 2020). Therapeutic oncology vaccines have been on the developmental road for decades; however, their progress has been slowed by setbacks and failures. With technological advances, however, great progress has been made in this research area. Among these vaccines, personalized neoantigen vaccines are the most eye-catching new technology (Blass & Ott, 2021). Owing to genetic mutations, tumor cells express large amounts of mutated proteins that are not expressed by normal cells. These mutated DNA and proteins in the form of a vaccine produce a precise immune response that targets only tumor cells (Hu et al., 2021). However, the DNA and protein antigens released by dead tumor cells alone are ineffective in activating dendritic cells (DCs) within the tumor to initiate a strong T-cell response (De Gregorio et al., 2013). Injecting nucleic acid adjuvants into the tumor can effectively activate the host’s antitumor immune responses. Exogenous DNA can activate DCs in tumors and then recruit and activate tumor-specific T cells to completely destroy tumor cells (Krieg et al., 1998). Toll-like receptors (TLRs) are nucleic acid receptors distributed on the surface of immune cells or the endoplasmic reticulum. TLR activation initiates the transcription of genes that help amplify the immune response (Steinhagen et al., 2011). Checkmate Pharmaceuticals has been developing CMP-001 as an unmethylated cytosine-phosphate-guanine (CpG) oligodeoxynucleotide TLR9 agonist, which is delivered with a virus-like particle (VLP) (Miller et al., 2021). Plasmacytoid DCs are immature DCs that infiltrate tumors and contribute to the immunosuppressive tumor microenvironment (TME). After VLPs bind to pDC surface receptors, CpG enters the cell interior, activates TLR9, increases the transcription of IFN-α and other genes, promotes the pDC presentation of tumor antigens to T cells, stimulates the expansion of T cells, recruits high-quality conventional DCs (cDCs), and ultimately enhances the antitumor response. In addition, the induction of tumor ICD *via* radiotherapy or chemotherapy can also activate the T-cell immune response by releasing large amounts of tumor antigens and signaling molecules (Zhu et al., 2021), including calreticulin (CRT) exposed on the cell surface, high mobility group protein 1 (HMGB1), and ATP molecules. These signaling molecules can bind to pattern-recognition receptors on the DC cell surface, initiate specific cytological responses, and ultimately activate innate and adaptive immune responses (Rapoport and Anderson, 2019).

T-cell invasion in the tumor tissue can be promoted by dredging tumor blood vessels (Bourhis et al., 2021). Tumor blood vessels are abnormal, characterized by disorganized and leaky walls, elevated interstitial hydraulic pressure, poor hemoperfusion, and poor drug delivery ability (Feng et al., 2021). In 2013, Rakesh Jain introduced the concept of vascular normalization, which advocated low-dose antiangiogenic drugs such as bevacizumab and ramucirumab to normalize tumor blood vessels so that they were evenly distributed within the tumor, thereby facilitating the infiltration of effector T cells (Huang et al., 2013). The combination of atezolizumab (anti-PD-L1) and bevacizumab (anti-VEGF) produced surprising phase III clinical trial results in unresectable hepatocellular carcinoma (HCC). In terms of overall survival (OS) and progression-free survival (PFS), combination therapy was significantly superior to sorafenib, the first-line therapy for hepatocellular carcinoma (ClinicalTrials.gov Identifiers: NCT04770896, NCT04712643, NCT04732286). Recently, combination therapy based on vascular endothelial growth factor (VEGF)/VEGF receptor has become one of the most sought-after targets for the clinical development of antitumor drugs (Ren et al., 2021). After infiltrating into tumors, CD8^+^ T cells have to overcome the adverse immunosuppressive microenvironment and they are often exhausted after long-term antigen stimulation under these adverse conditions. These exhausted CD8^+^ T cells cannot efficiently proliferate, produce cytokines, and destroy tumor cells (Miller et al., 2019). This is mainly because of the upregulation of several inhibitory receptors, including PD-1, CTLA-4, TIM-3, and LAG-3 (Anderson et al., 2016; Kim et al., 2021; Li et al., 2022a; Li et al., 2022b; Cui, 2022). These inhibitory receptors bind to ligands on the surface of APCs or tumor cells to further inhibit the immune function of T cells (Ma et al., 2019). Therefore, blocking coinhibitory receptors can reverse T cell depletion and free their ability to kill tumor cells.

**SCHEME 1:**
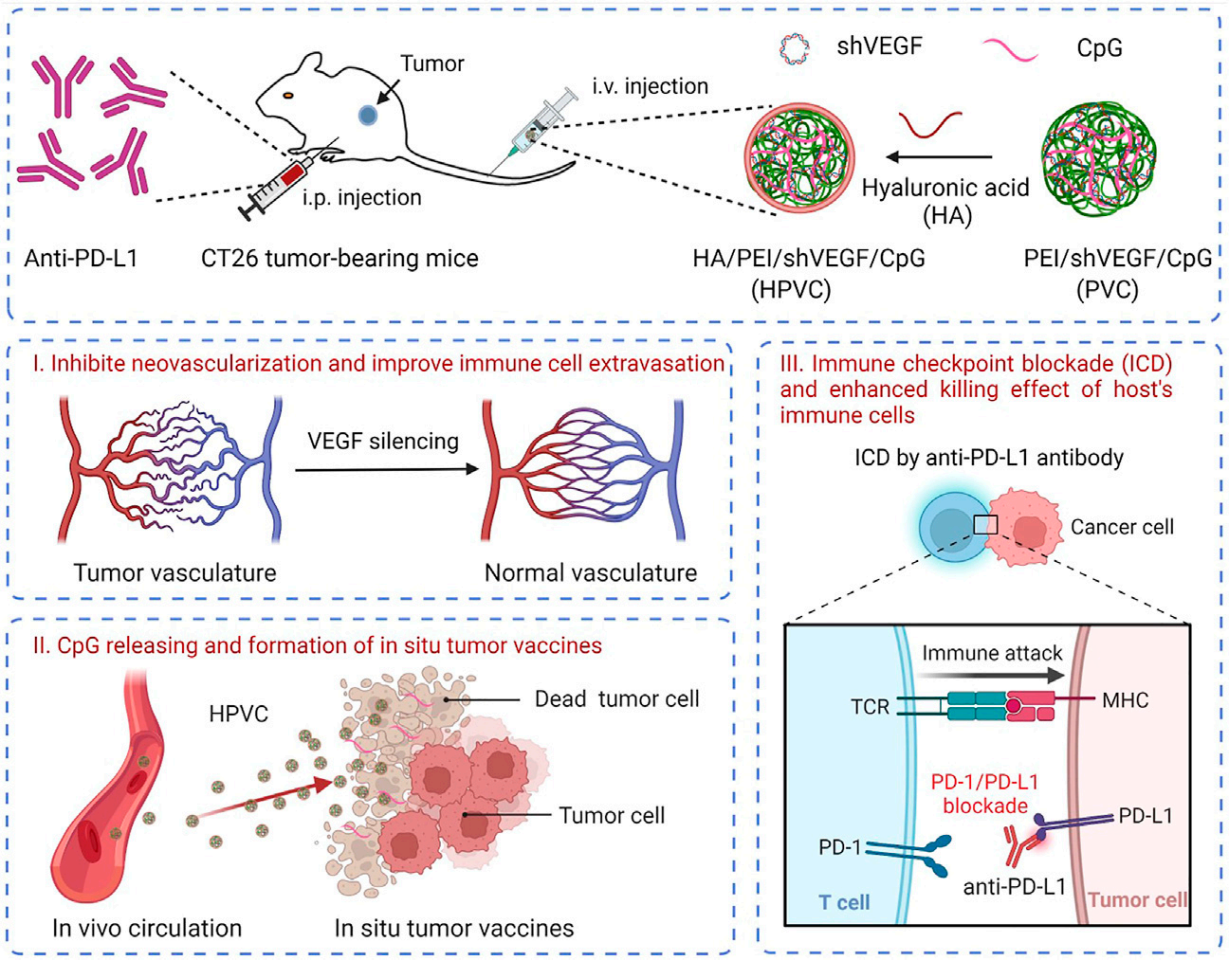
Schematic illustration of the combination therapy of HA/PEI/shVEGF/CpG targeted gene therapy of anti-angiogenesis and PD-1/PD-L1 blockade for facilitating antitumour immunotherapy. Created with BioRender.com.

Tumor immunotherapy can improve the antitumor therapeutic effect by simultaneously activating effector T cells, facilitating tumor tissue infiltration, and activating immune-mediated tumor killing. In the present study, a synergistic strategy was developed by combining *in situ* tumor vaccines, gene-mediated anti-angiogenesis and anti-PD-L1 therapy. Unmethylated CpG and VEGF-silencing gene (shVEGF) were co-delivered *via* a hyaluronic acid (HA)-modified HA/PEI/shVEGF/CpG system to trigger *in situ* tumor vaccine and antiangiogenic effects. To further improve this specific tumor killing effect, an anti-PD-L1 antibody was introduced to achieve immune checkpoint blockade, thereby boosting antitumor immune responses (Scheme 1). This strategy had the following advantages. 1) VEGF silencing in the tumor tissue could reduce tumor angiogenesis, facilitating tumor cell infiltration. 2) Necrotic tumor cells and CpG adjuvants could form an *in situ* tumor vaccine and activate the host immune response. 3) PD-1/PD-L1 blockade could further activate the antitumor killing ability of T cells and improve the antitumor efficacy. Thus, the combination therapy strategy enabled the design of multiple stages in the tumor immunotherapy cycle and is expected to provide a new avenue for the clinical treatment of tumors.

## 2 Materials and methods

### 2.1 Materials

Hyperbranched polyethyleneimine (25 kDa, PEI25k) was ordered from Sigma‒Aldrich (United States). The plasmid DNA extraction and protein detection kits were obtained from Tiangen Biotech (Beijing) Co., Ltd. (Beijing, China). Luciferase reporter gene assay kit were purchased from Promega (Mannheim, Germany). MTT was purchased from Sigma‒Aldrich (United States). Hyaluronic acid (HA, ∼40 KDa) was ordered from Freda (Shandong, China). CpG oligonucleotides (5′-TCC​ATG​ACG​TTC​CTG​ACG​TT-3′) were ordered from Sangon Biotech (Shanghai, China). A plasmid that encoded the corresponding small hairpin RNA targeting the VEGF gene (shVEGF) was constructed by GenePharma (Suzhou, China), and the coding region of the VEGF gene was 5′-AUG​UGA​AUG​CAG​ACC​AAA​GAA-3’. RNA extraction kits, reverse transcription kits and real-time quantitative polymerase chain reaction (RT‒qPCR) kits were ordered from Tiangen Biotech (Beijing) Co., Ltd. Anti-mouse PD-L1 antibody was ordered from BioXcell, Inc. (West Lebanon, United States). All antibodies were purchased from eBioscience (CA, United States).

### 2.2 Preparation of PEI/shVEGF/CpG nanoparticles

PEI/shVEGF/CpG nanoparticles were obtained by electrostatic incorporation. Briefly, PEI25k, shVEGF, CpG and HA were dissolved to 1 mg/mL in distilled water. PEI/shVEGF/CpG (PVC) nanoparticles were obtained by simply mixing at a mass ratio of 2:1:0.5. After vortexing for 30 s, the PVC nanoparticles were incubated for 25 min at room temperature (RT). HA/PEI/shVEGF/CpG (HPVC) nanoparticles were further prepared by introducing different amounts (0.25:2:1:0.5, 0.5:2:1:0.5, 0.75:2:1:0.5 and 1:2:1:0.5) of HA to PVC nanoparticle solutions. After vortexing for 30 s, HPVC nanoparticles were harvested by incubating at RT for 25 min.

### 2.3 Particle sizes and zeta potentials

HA/PEI/shVEGF/CpG (HPVC) nanoparticles with different mass ratios (0:2:1:0.5, 0.25:2:1:0.5, 0.5:2:1:0.5, 0.75:2:1:0.5 and 1:2:1:0.5) were prepared as previously mentioned. The concentration of CpG was 0.05 mg/mL. The particle sizes and zeta potentials of HPVC and PVC were detected *via* a zeta potential/BI-90Plus particle size analyzer (Brookhaven, United States). The morphology was further observed by transmission electron microscopy (TEM). Briefly, HPVC solutions (0.02 mg/mL of shVEGF) was dropped onto a 200 mesh copper grid coated with carbon and dried at room temperature. The TEM figures were observed *via* an electron microscope operating at an acceleration voltage of 100 kV (JEOL JEM-1011, Japan).

### 2.4 Stability of HPVC nanoparticles

HPVC or PVC nanoparticles were prepared and incubated in phosphate-buffered saline (PBS, pH 7.4) containing bovine serum albumin (BSA, 0.25 mg/mL). The final concentration of CpG was 0.05 mg/mL. The stability was evaluated by monitoring the particle size of HPVC nanoparticles at different incubation times.

### 2.5 Cell culture and cell viability

Murine colorectal adenocarcinoma cells (CT26) were incubated in DMEM containing FBS (10% v/v), penicillin (100 U mL^-1^) and streptomycin (100 mg mL^-1^). For the cell viability assay, the cells were seeded in 96-well plates (1 × 104 cells/well) and incubated overnight. Different amounts of HA/PEI/pDNA/CpG nanoparticle solutions were added to the wells. After 24 h, 20 μl of MTT solution (5 mg mL^-1^ in PBS) was added and incubated for 3 h. The solutions were carefully removed from each well, and 200 μl of DMSO was added. The absorption was detected at 492 nm by a microplate reader (TECAN, Switzerland). The absorbance value of the well without any treatments was considered to be 100%, and the relative cell viability was obtained by calculation.

### 2.6 Gene transfection

The optimum transfection condition was first evaluated by the luciferase reporter gene (pGL3). Briefly, CT26 cells (1×10^4^ cells/well) were seeded in 96-well plates and incubated overnight. HA/PEI/pGL3/CpG nanoparticle solutions with various mass ratios of HA and CpG were added, and the final concentration of pGL3 was 1 μg/mL. After 48 h, the supernatants were removed, and the cells were washed twice with PBS solution. Cell lysate (50 µl/well) was added, and the 96-well plates were frozen at −80°C for 1 h. After thawing, the relative luciferase units (RLU) were monitored after mixing with luciferase substrates *via* a luminometer (Promega, United States), and the total protein was detected by protein quantitative detection kits (Thermo Fisher Scientific, United States). The gene transfection efficiency was evaluated as RLU/mg protein.

The quantitative expression of VEGF mRNA was measured by RT‒qPCR at the optimum transfection condition of HA/PEI/shVEGF/CpG nanoparticles. Briefly, CT26 cells were seeded in 6-well plates (1.5 × 10^5^ cells/well) and incubated overnight. HA/PEI/shVEGF/CpG, PEI/shVEGF/CpG and shVEGF were added to the wells. The final concentration of shVEGF was 1 μg/mL. After 24 h, the total RNA was collected by an RNA extraction kit (TIANGEN, China). Then, reverse transcription and qPCR amplification were carried out by a quantitative PCR detection system (Stratagene, United States). The primers for the VEGF gene were as follows: forward, 5′-GGT GAG AGG TCT AGT TCC CGA-3’; reverse: 5′-CCA TGA ACT TTC TGC TCT TC-3’. For GAPDH: Forward, 5′-GTT CCA GTA TGA CTC TAC CC-3’; Reverse, 5′-AGT CTT CTG AGG CAG TGA TG-3’. The secretion of VEGF protein in the supernatant 48 h post transfection in CT26 cells was detected using a VEGF ELISA kit. The OD values were measured at 450 nm, and the VEGF protein content was calculated according to the standard curve.

### 2.7 Intracellular uptake

The cell endocytosis of HPVC nanoparticles was evaluated by flow cytometry. Briefly, 5×10^4−ΔΔCT^26 cells were seeded in 24-well plates and incubated overnight. HA/PEI/FAM-DNA/CpG, PEI/FAM-DNA/CpG and FAM-DNA solutions were added and incubated for 3 h. FAM fluorescence-labelled DNA (FAM-DNA) was used (1 μg/mL) as a tracer. Then, the cells were digested, collected and measured by a flow cytometer (FACSCelesta, BD, United States).

The intracellular uptake of HPVC nanoparticles was further observed by confocal laser scanning microscopy (CLSM). Briefly, 1.5×10^5−ΔΔCT^26 cells were seeded in 6-well plates with a cover glass in advance and incubated overnight. HA/PEI/FAM-DNA/CpG, PEI/FAM-DNA/CpG and FAM-DNA solutions were added and incubated for 3 h. Then, the wells were washed with cold PBS and fixed with paraformaldehyde (4%, v/v) for 10 min at RT. The nuclei were stained with DAPI (1 μg/mL) for 10 min and mounted with glycerimum. Finally, the samples were observed *via* CLSM (ZEISS780, Germany).

### 2.8 Differentiation of BMDCs

Bone marrow-derived dendritic cells (BMDCs) were extracted from Balb/c mice (male, 6 weeks old). Briefly, the mice were sacrificed and disinfected in 75% (v/v) alcohol for 30 min. Then, the femurs and tibias were excised. After sterilization and rinsing, the ends of the bones were cut off, and the bone marrow cells were washed out with a needle. The cell suspension was collected, and the precipitated cells were harvested after centrifugation at 1500 rpm for 4 min and then resuspended in RPMI 1640 medium supplemented with 10% FBS, 20 ng/mL recombinant mouse GM-CSF and 10 ng/mL IL-4. Replace 1/2 volume of fresh medium every 2 days. On the sixth day, BMDCs were harvested.

### 2.9 Activation of BMDCs

The activation of BMDCs was evaluated *via* a transwell assay. CT26 cells were seeded in the upper wells at 5 × 10^5^ cells per well and incubated overnight. HA, PEI25k, shVEGF, CpG, HA/PEI/shVEGF and HA/PEI/shVEGF/CpG solutions were added to the upper wells; the mass ratio of HA/PEI/shVEGF/CpG nanoparticles was 1:4:2:1 (0.5 μg/mL of CpG). After 48 h, the upper wells and the medium were transferred to the new lower wells precultured with BMDCs (5 × 10^5^ cells/well). After 24 h, the supernatant was collected to analyze IL-12p70 and TNF-α levels *via* enzyme-linked immunosorbent assay (ELISA). BMDC maturation was further evaluated by treatment with FITC-conjugated anti-CD11c, PE-conjugated anti-CD80 and APC-conjugated anti-MHCII antibodies for 45 min at 4°C. Finally, the cells were detected *via* flow cytometry and analyzed using FlowJo V10 software.

### 2.10 Antitumor study

BALB/c mice (female, 16–18 weeks, 18–20 g) were ordered from Beijing Vital River Laboratory Animal Technology Co., Ltd. The animal procedures were approved in the guidelines established by the Animal Care and Use Committee of Jilin University. Briefly, tumor-bearing mice were constructed by subcutaneous injection of 1.0×10^6−ΔΔCT^26 cells on day 0. When tumor volumes were approximately 80 mm^3^, the mice were injected with PBS, anti-PD-L1 antibody, HA/PEI/shVEGF/CpG, and HA/PEI/shVEGF/CpG + anti-PD-L1 on days 8, 11, 14 and 17 post tumor inoculation. HPVC nanoparticles and anti-PD-L1 antibody were injected into the mice *via* intravenous injection and intraperitoneal injection, respectively. The single injection dose was 12.5 μg HA, 50 μg PEI25k, 25 μg shVEGF, 12.5 μg CpG and 50 μg anti-PD-L1. The tumor sizes and body weights of the mice were monitored every 2 days. The tumor volumes were calculated as follows: tumor volume V) = length × (width)^2^/2. The tumor suppression rate (TSR) was calculated as follows: TSR (%) = [(Vc - Vt)/Vc] × 100%, where Vc and Vt represent the average tumor volume of the PBS control group and treatment group, respectively. At day 19, the mice were sacrificed, and then the tumor tissues, main organs, lymph nodes and serum samples were further evaluated.

### 2.11 Tumor rechallenge assay

Naïve Balb/c mice and HA/PEI/shVEGF/CpG + anti-PD-L1-treated Balb/c mice from antitumor therapy experiments (day 19) were subcutaneously inoculated (the other side) with 1 × 10^6−ΔΔCT^26 cells. The tumor volumes of the mice were detected and recorded every 2 days.

### 2.12 Detection of immune cells and cytokines

To investigate the mechanism of immunotherapy, immune cells were analyzed after the antitumor study. Briefly, the tumors, kidneys and lymph nodes were collected after treatment. The tissues were cut into small pieces and filtered through 300 mesh nylon filters to harvest single-cell suspensions. Lymph node and splenic single cells were stained with fluorescent antibodies for DC activation detection and TEM cells. Tumor-infiltrating CD4^+^ T cells, CD8^+^ T cells, M1 macrophages, NK cells, MDSCs, M2 macrophages, and Treg cells. Then, the cells were washed with cold PBS and analyzed by flow cytometry (BD, United States). The cytokines (TNF-α, IFN-γ, TGF-β and IL-10) secreted in serum were detected by ELISA kits.

### 2.13 H&E and immunofluorescence

After treatment, the tumors and main organs (heart, liver, spleen, lung and kidney) were excised and fixed in 4% paraformaldehyde. Then, the samples were embedded in paraffin and sectioned. The slices were subjected to histopathological H&E staining. In addition, the contents of CD8^+^ T cells and CD4^+^ T cells in tumors were analyzed by immunofluorescence. Briefly, the tumor slices were stained with sheep anti-mouse CD8 and rabbit anti-mouse CD4 monoclonal antibodies. Then, AF488-labelled donkey anti-sheep and Cy3-labelled goat anti-rabbit secondary antibodies were added. Finally, the samples were detected by CLSM (Zeiss780, Germany).

### 2.14 *In vivo* safety evaluation

After the antitumor study, the mice were euthanized by isoflurane (air mixing percentage 1.5%, air mass flow 2 L/min) *via* a mouse anesthesia system (RWD, Shenzhen, China), and the serum was collected by centrifugation for 10 min at 10,000 rpm. Then, the representative indicators of liver (ALP, ALT and AST) and kidney (CRE, UA and BUN) function indexes were measured by ELISA kits.

### 2.15 VEGF gene expression in tumors

The expression of VEGF mRNA in tumors was measured using RT‒qPCR assay. After antitumor treatment, the tumors were extracted and cut into small pieces. Total RNA was extracted using an RNA extraction kit (TIANGEN, China). After quantification, the total RNA was reverse transcribed to cDNA. RT-qPCR was performed using a quantitative PCR detection system (Stratagene, United States) according to the manufacturer’s instructions. Furthermore, the VEGF protein level in the tumor tissues was measured *via* ELISA. In brief, the tumor tissues were homogenized and lysed, and the supernatants were collected and analyzed using a VEGF ELISA kit. The OD values were measured at 450 nm, and the VEGF level content was calculated according to the standard curve.

### 2.16 Statistical analysis

All experiments were detected at three or more replicate samples and illustrated as the means ± standard deviations (s.d.). Flow cytometry results were performed using FlowJo V10 software. Statistical analysis was carried out using the unpaired two-tailed Student’s t-test in GraphPad Prism eight software.

## 3 Results and discussion

### 3.1 Preparation and characterization of HA/PEI/shVEGF/CpG (HPVC) nanoparticles

**FIGURE 1:**
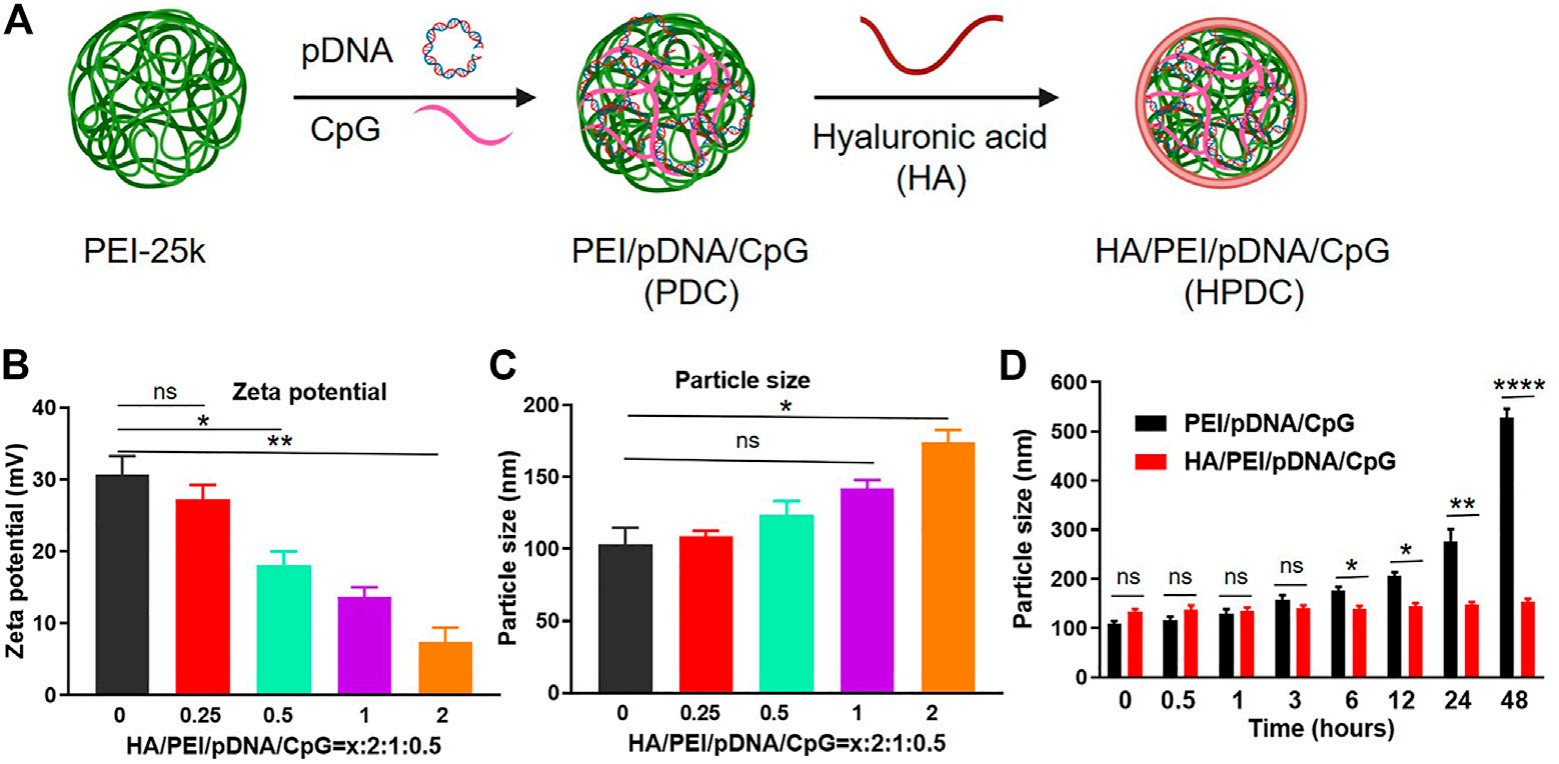
Preparation and characterization of HA/PEI/pDNA/CpG nanoparticles. **(A)** Preparation route of HA/PEI/pDNA/CpG nanoparticles. **(B)** Zeta potentials and **(C)** particle sizes of HA/PEI/pDNA/CpG nanoparticles at different mass ratios. **(D)** Stability of HA/PEI/pDNA/CpG nanoparticles in FBS solutions at different incubation time points. ns = no significance, *p < 0.05, **p < 0.01, ****p < 0.0001.

HPVC nanoparticles were constructed *via* simple electrostatic incorporation (Figure 1A). The effect of HA with different mass ratios on the performance of the gene delivery system (PEI/shVEGF/CpG, PVC) was evaluated *via* zeta potentials (Figure 1B) and particle sizes (Figure 1C). The results revealed that this simple strategy could form nanoparticles and that the zeta potentials decreased with increasing HA amount, which can be attributed to the induction of negative HA molecules. The particle size of HPVC nanoparticles enlarged possibly *via* the excessive negative charge attracting the positive charge of the nanoparticles. The morphology of HPVC nanoparticles was observed using TEM, and the result demonstrated that HPVC nanoparticles exhibited a spherical morphology, approximately 100 nm in diameter (Supplementary Figure S1). To further evaluate the stability of HPVC nanoparticles, the nanoparticles were incubated in FBS solutions for various incubation times. As shown in Figure 1D, the introduction of HA increased the stability of HPVC nanoparticles compared with that of PVC nanoparticles owing to the reduced adsorption of nanoparticles and serum proteins after introducing HA molecules. These properties made the *in vivo* application of the prepared delivery system more conducive.

### 3.2 Characterization of HPVC nanoparticles in CT26 cells

**FIGURE 2:**
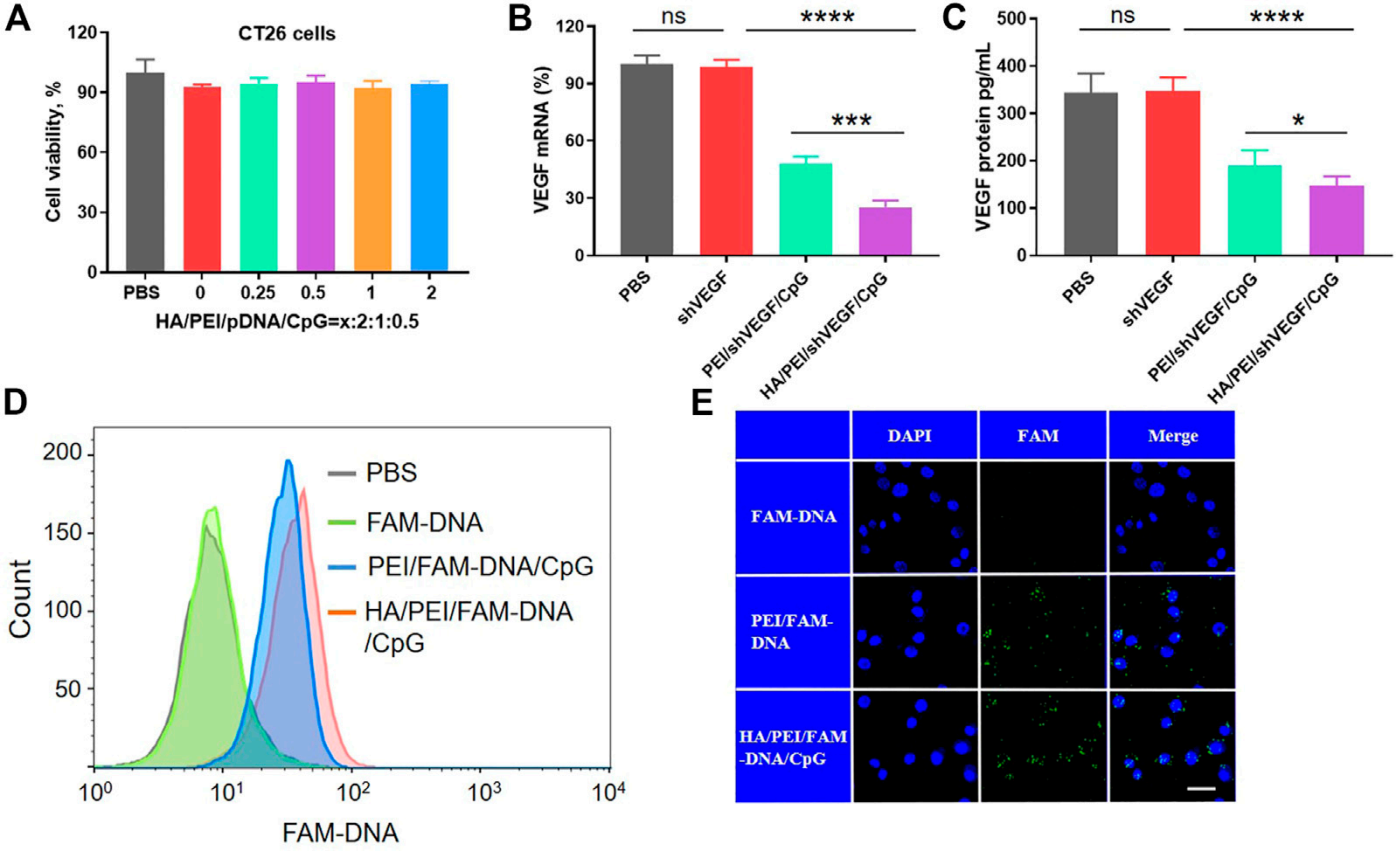
Cellular characterization of HA/PEI/pDNA/CpG nanoparticles in CT26 cells. **(A)** Cell viability of HA/PEI/pDNA/CpG nanoparticles at various contents of HA. **(B)** VEGF mRNA expression after gene transfection by RT‒qPCR assay. **(C)** The secretion of VEGF levels in culture medium were measured by ELISA kit 48 h after transfection in CT26 cells. **(D)** Cell endocytosis efficiency of HPDC, PDC and FAM-DNA after 3 h of incubation in CT26 cells by flow cytometric analysis. **(E)** The intracellular uptake of HPDC, PDC and FAM-DNA after 3 h of incubation in CT26 cells by CLSM. The scale bar is 20 μm ns = no significance, *p < 0.05, **p < 0.01.

Good cytocompatibility is a prerequisite for the successful application of biomedical materials *in vivo*. In general, cationic groups, which can interact with negatively charged components on the target cell membrane and induce membrane destabilization, produce the cytotoxicity of gene delivery systems. The cytotoxicity of HPVC nanoparticles was measured at various mass ratios, and the results (Figure 2A) revealed no obvious cytotoxicity under laboratory conditions, indicating the potential of HPVC nanoparticles for clinical application.

Next, the transfection efficiency of HPVC nanoparticles was evaluated in CT26 cells. The optimal transfection additions were screened using the luciferase reporter gene. The results demonstrated that HPVC nanoparticles achieved the highest gene transfection efficiency at a mass ratio of 0.5:2:1:0.5 (Supplementary Figure S2 and Supplementary Figure S3). To evaluate the biological function, the gene silencing efficiencies of PVC and HPVC nanoparticles were detected by evaluating the expression of VEGF mRNA by using RT-qPCR after 24 h of transfection. The results showed that shVEGF itself could hardly induce a gene-silencing effect; the PVC group exhibited an approximately 52.1% silencing effect, whereas the effect in HPVC groups was 74.7% (Figure 2B). The expression of VEGF protein in the supernatants was detected using ELISA, and the results showed a trend consistent with the mRNA detection results: HPVC group exhibited the highest silencing effect than the other groups (Figure 2C). These results were consistent with previous results and further demonstrated the effectiveness of HA.

In general, increasing the electronegative properties of the gene delivery system reduces the efficiency of endocytosis (Chen et al., 2022). In the current study, the surface charges of HPVC nanoparticles gradually decreased with increasing HA content. In theory, their endocytosis efficiency should have been reduced, but an opposite effect was observed (Figure 2D). This effect can be attributed to the overexpression of CD44, which are receptors that specifically bind HA on CT26 cells (Seok et al., 2018). To further evaluate the targeting capacity of HA to CT26 cells, the intracellular uptake of HPVC nanoparticles was observed *via* CLSM, with FAM-DNA was utilized as the fluorescent signal. As shown in Figure 2E, relatively higher intracellular uptake was observed in HPVC nanoparticles than in PVC nanoparticles, which was consistent with the previous flow cytometry results.

### 3.3 Activation of BMDCs

**FIGURE 3:**
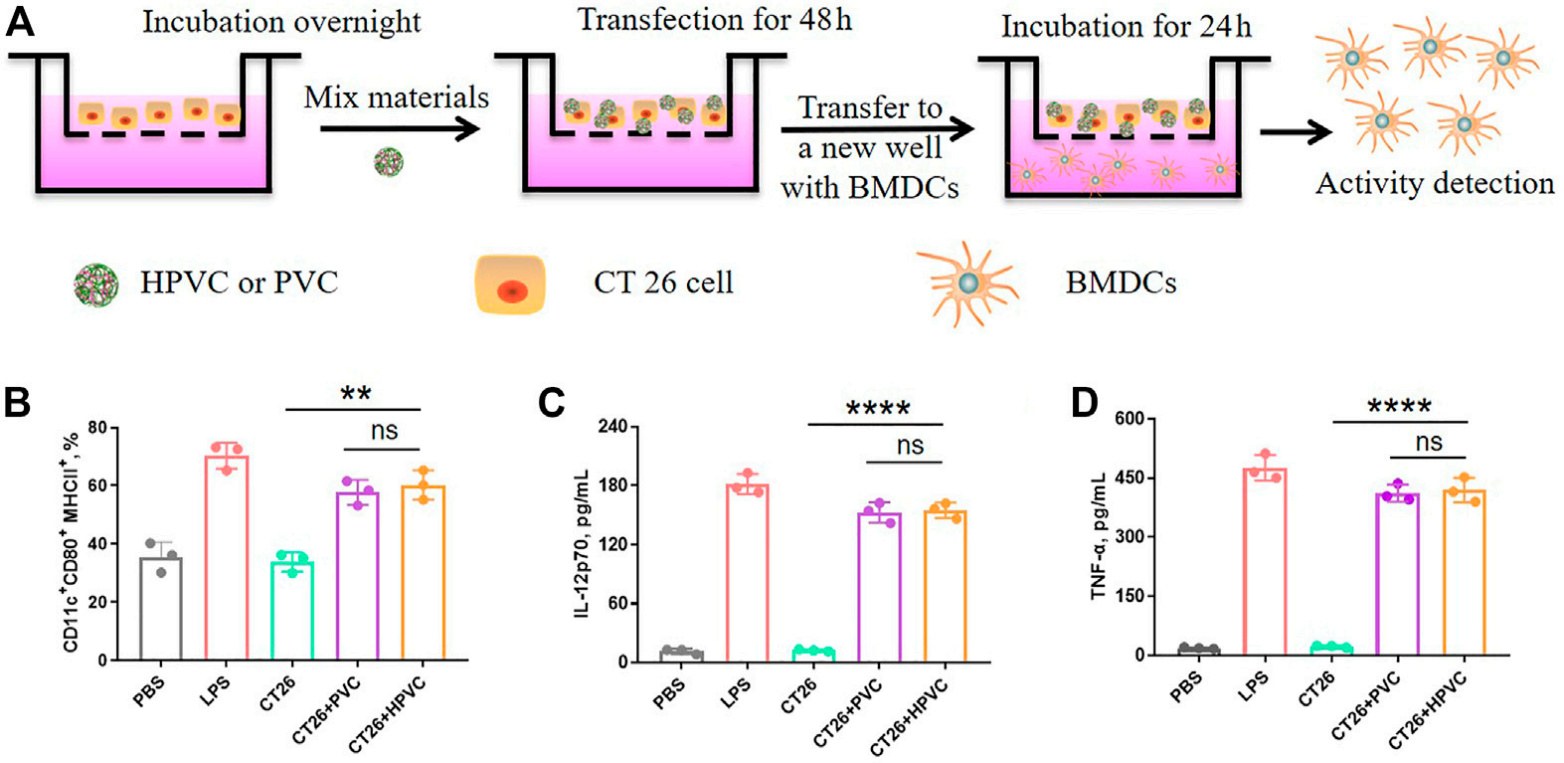
*In vitro* activation of BMDCs by transwell assay. **(A)** Strategy for BMDC activation *in vitro*. **(B)** BMDC maturation gating on CD11c^+^CD80^+^MHCII^+^ cells. **(C)** IL-12p70 and **(D)** TNF-α cytokine secretion in the medium. ns = no significance, **p < 0.01.

To mimic the immune activation of HPVC nanoparticles *in vivo*, the maturation of BMDCs was detected *in vitro* using a transwell assay (Figure 3A). CT26 cells in the upper wells were transfected with HPVC or PVC nanoparticles for 48 h. The upper wells and the culture medium were then transferred to the new lower wells preincubated with BMDCs and co-incubated for 24 h. BMDCs were then collected for evaluating maturation rates. As shown in Figure 3B, CT26 cells without any treatment revealed a maturation ratio comparable to that of the PBS control group (35.6%), indicating the poor activation capacity of BMDCs. The HPVC and PVC nanoparticle groups produced 60.3% and 57.8% BMDC maturation rates, respectively. No significant differences were observed between these two groups. This was mainly owing to the introduction of unmethylated CpG immune agonists and CpG oligonucleotide agonists that activate BMDCs. The supernatants were collected to evaluate the cytokine secretion of mature BMDCs, and the results revealed that IL-12p70 (Figure 3C) and TNF-α (Figure 3D) levels were significantly increased after incubation with HPVC and PVC nanoparticles, further indicating their effective immune activation.

### 3.4 Antitumor therapy using HPVC

**FIGURE 4:**
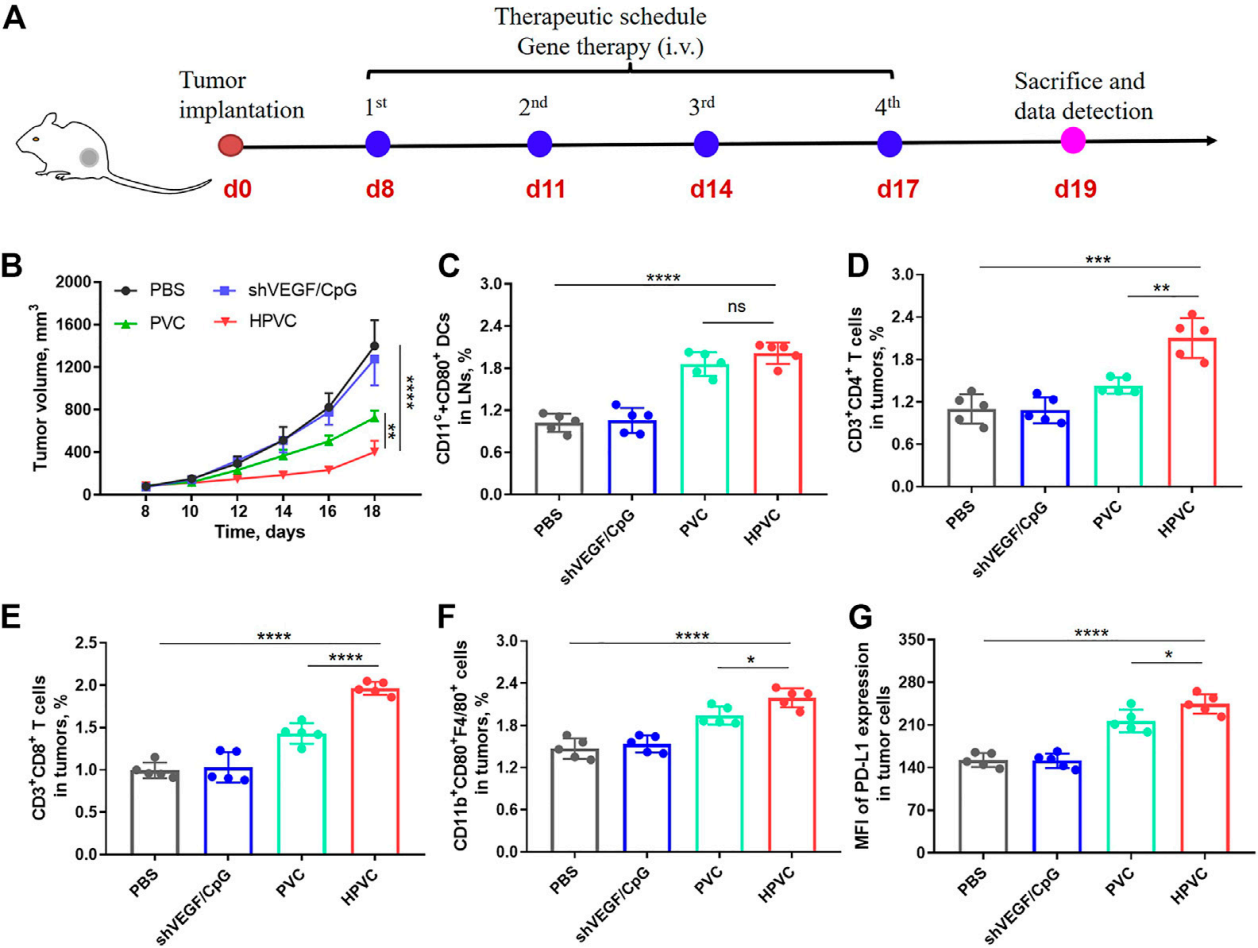
Antitumor therapy by HPVC. **(A)** Schematic illustration of antitumor therapy of CT26 tumor-bearing mice by HPVC. **(B)** Average tumor growth curves of mice in different treatment groups (*n* = 5). **(C)** Flow cytometry analysis of DC maturation in LNs. Flow cytometry analysis of **(D)** CD3^+^CD4^+^ T cells, **(E)** CD3^+^CD8^+^ T cells and **(F)** CD11b^+^CD80^+^F4/80^+^ macrophages. **(G)** Mean fluorescence intensity (MFI) of PD-L1 expression in tumor cells. ns = no significance, *p < 0.05, **p < 0.01, ***p < 0.001, ****p < 0.0001.

Encouraged by the high gene transfection efficiency and immune activation results of HPVC nanoparticles, the therapeutic effect of HPVC nanoparticles was evaluated in a CT26 tumor model. PBS, free shVEGF/CpG, PVC and HPVC were administered intravenously at 8, 11, 14, and 17 days (Figure 4A). The naked shVEGF/CpG group showed a negligible effect in inhibiting CT26 tumor growth, which was mainly caused by its rapid removal of nuclease from the host after intravenous injection (Figure 4B). The PVC group exhibited a modest inhibition effect (48.3%) compared with that of the HPVC group (71.2%) on day 18. In the preliminary experiment, we performed antitumor experiments using non-therapeutic pDNA and CpG mimics (mCpG) with disrupted nucleic acid sequence. The results showed that the HA/PEI delivery system had no tumor suppressive effect when delivering non-therapeutic pDNA and mCpG mimics compared with the PBS control group (Supplementary Figure S4A). In addition, there were no obvious differences in body weights for any of these two groups (Supplementary Figure S4B). Therefore, the effective antitumor effect of HPVC nanoparticles was primarily derived from the highly efficient delivery system, shVEGF therapy gene, and CpG agonists. The CT26 tumor-targeting delivery system achieved efficient delivery and gene-silencing effects in the tumor tissues. CpG agonists activated APCs and achieved significant ampliative maturation of DCs (Figure 4C). VEGF downregulation *via* shVEGF therapy regulated the tumor microenvironment and facilitated the infiltration of immune cells, including CD3^+^CD4^+^ T cells (Figure 4D), CD3^+^CD8^+^ T cells (Figure 4E) and CD11b^+^CD80^+^F4/80^+^ macrophages (Figure 4F), which was consistent with previously reported findings. (Huang et al., 2013). The detailed T-cell gating strategies using FlowJo V10 software are shown in Supplementary Figure S5. However, tumor growth increased obviously at the last stage of HPVC treatment (Figure 4B). The high expression of immunosuppressive molecules on tumor cells is the main factor in tumor immune escape, which leads to poor therapeutic effects. The inhibitory molecule expression on tumor cells was analyzed, and the results showed that HPVC treatment resulted in increased PD-L1 expression on CT26 tumor cells (Figure 4G). PD-L1 molecules bind to inhibitory receptors (PD-1) on the T-cell surface and inhibit its lethal effect. Therefore, blocking coinhibitory receptors can reverse T cell depletion and enable their tumor-killing ability.

### 3.5 Combined antitumor therapy with HPVC and anti-PD-L1 antibody

**FIGURE 5:**
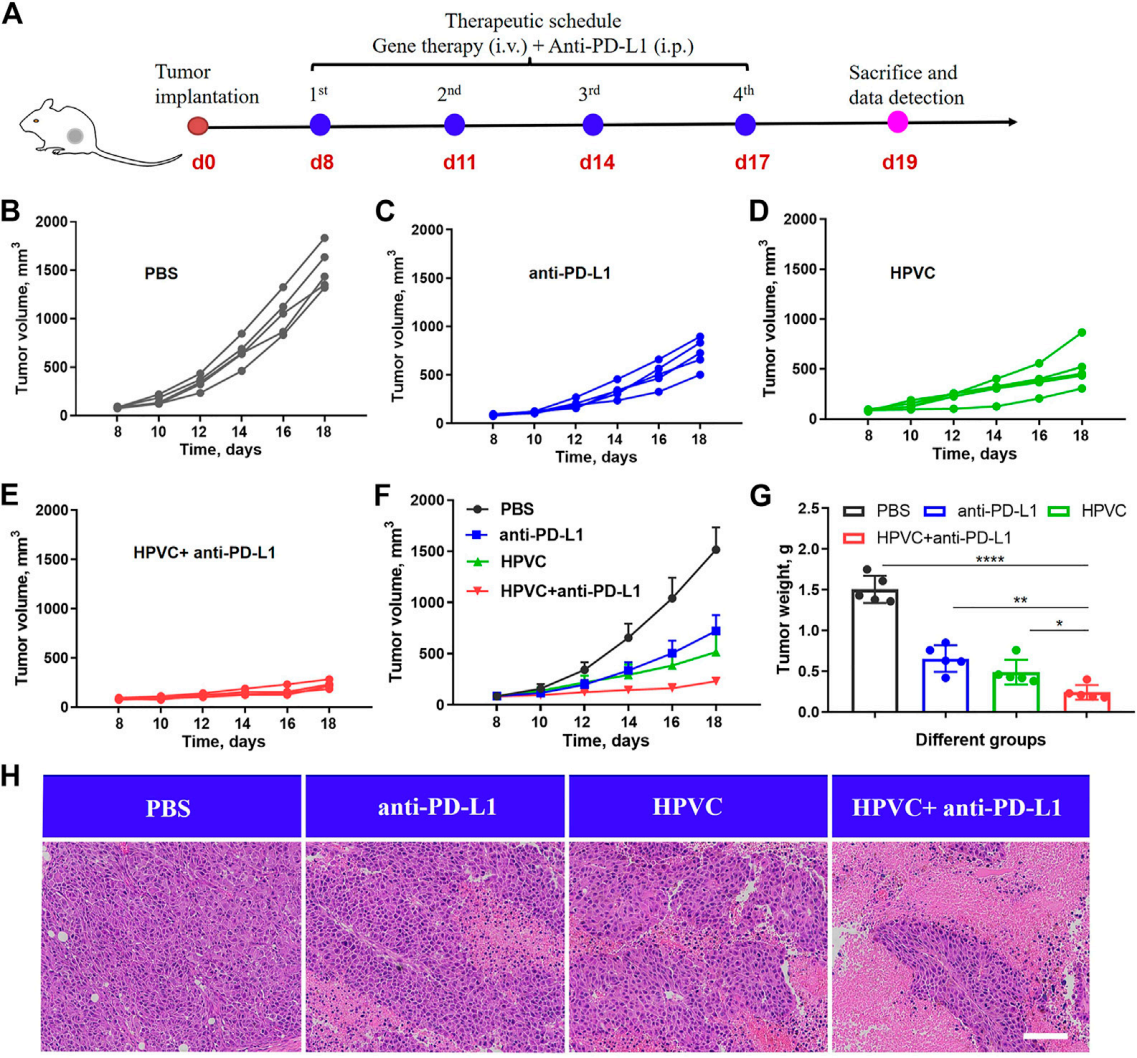
Combined antitumor therapy by HPVC and anti-PD-L1 antibody. **(A)** Schematic illustration of antitumor therapy in CT26 tumor-bearing mice by combined therapy. Individual tumor growth curves of mice in **(B)** PBS, **(C)** anti-PD-L1, **(D)** HPVC and **(E)** anti-PD-L1+HPVC. **(F)** Average tumor growth curves of mice in different treatment groups (n = 5). **(G)** Average tumor weight in different groups (n = 5). **(H)** H&E histological analysis of tumor tissues on day 19. Scale bar = 200 μm *p < 0.05, **p < 0.01, ****p < 0.0001.

To overcome the immune tolerance of tumor cells, an anti-PD-L1 antibody was introduced to achieve immune checkpoint blockade. As shown in Figure 5A, CT26 tumor models were constructed at day 0, and PBS, anti-PD-L1 antibody, and HPVC + anti-PD-L1 were administered at 8, 11, 14, and 17 days (Figure 5A). HPVC and anti-PD-L1 were administered *via* intravenous and intraperitoneal routes, respectively. The tumor sizes and body weights of the mice were monitored every 2 days. Average tumor growth curves (Figures 5B–F) demonstrated that both the anti-PD-L1 (Figure 5C) and HPVC (Figure 5D) groups exhibited a modest effect on inhibiting CT26 tumor growth. The combination treatment group (anti-PD-L1+HPVC) showed the best tumor suppression rate of 84.2% on day 18 (Figure 5E and F). The results of tumor weight after excision further validated the effective antitumor proliferation ability of the combination treatment strategy (Figure 5G). Histological H&E analysis was evaluated to illustrate an intuitive representation of the antitumor effect revealed that the largest necrotic area in the tumor tissues were observed after combining HPVC and anti-PD-L1 antibody-induced immune checkpoint blockade therapy (Figure 5H).

### 3.6 Antitumor mechanism

**FIGURE 6:**
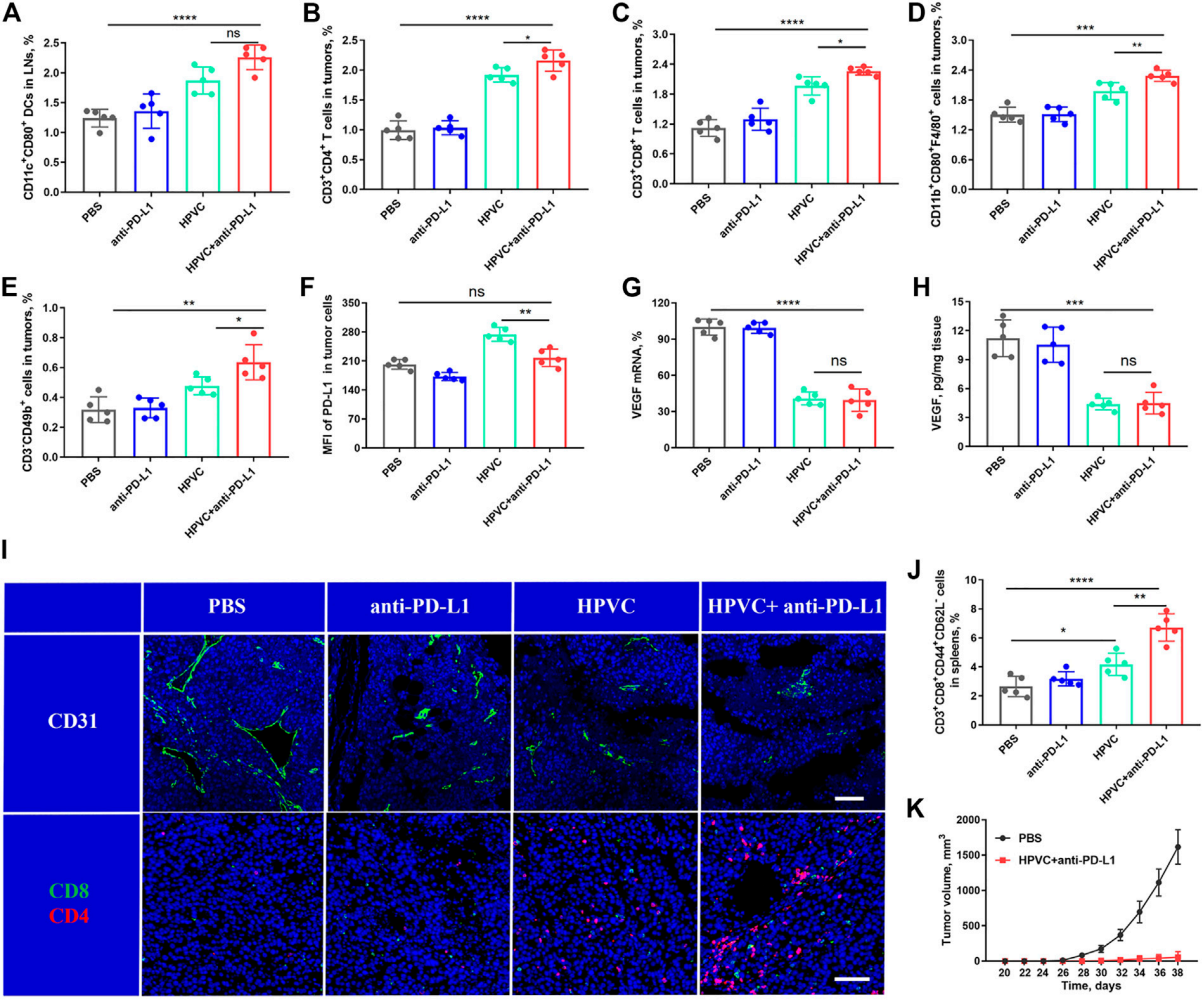
Analysis of immune cells after treatment. **(A)** DC maturation in LNs. **(B)** CD3^+^CD4^+^ T cells in tumors. **(C)** CD3^+^CD8^+^ T cells in tumors. **(D)** CD11b^+^CD80^+^F4/80^+^ macrophages in tumors. **(E)** CD3^−^CD49b^+^ NK cells in tumors. **(F)** Mean fluorescence intensity (MFI) of PD-L1 expression in tumor cells. **(G)** VEGF mRNA expression in tumors on day 19 by RT‒qPCR assay. **(H)** VEGF protein expression in tumors on day 19 by ELISA. **(I)** Representative images of CD31^+^ (green), CD8^+^ T cells (green) and CD4^+^ T cells (red) by immunofluorescence staining. Nuclei were stained with DAPI (blue). The scale bar is 200 μm. **(J)** TEM cells in spleens. **(K)** Average tumor growth curves of rechallenged tumors inoculated on day 19 by treatment with HPVC combined with anti-PD-L1 antibody (*n* = 5). *p < 0.05, **p < 0.01, ***p < 0.001, ****p < 0.0001.

The significant antitumor effect of the combination therapy was produced through the specific activation of the host’s antitumor immune responses. To assess the activation of DCs after treatment, the draining inguinal LNs were excised and collected, labeled with anti-CD11c and anti-CD80 antibodies, and detected *via* flow cytometry detection. Consistent with the previous results, the HPVC group induced a stronger DC activation (1.87%) than the PBS group (1.24%). The mature DC ratio (2.26%) obviously improved after combination with anti-PD-L1, indicating the feasibility and potential of the combined strategy (Figure 6A). Then, we measured the infiltration of immune-activated cells in tumors was measured. The results revealed that all immune cell proportions, including CD4^+^ T cells, CD8^+^ T cells, M1 macrophages, and NK cells, improved after combination therapy with HPVC and anti-PD-L1 antibody (Figure 6E). More importantly, PD-L1 expression on tumor cells was markedly decreased after combined anti-PD-L1 antibody therapy, which was compatible with the PBS group (Figure 6F), indicating immunosuppression blockade. The antitumor immune response is a complex process and requires the synergistic action of multiple immune cells and cytokines. After treatment administration, the immune-activating cytokines TNF-α (Supplementary Figure S6) and IFN-γ (Supplementary Figure S7) in the serum were significantly increased in the HPVC + anti-PD-L1 group, whereas the immune-inhibiting cytokines TGF-β (Supplementary Figure S8) and IL-10 (Supplementary Figure S9) were decreased to a certain extent. The enhanced immune activation *via* the combination therapy was mainly attributed to the activation of immune systems as well as anti-angiogenesis induced by VEGF gene silencing. Therefore, VEGF mRNA expression levels in tumors after treatment were then measured using RT-qPCR. As anticipated, the combination group achieved approximately 60.5% VEGF mRNA downregulation compared with the PBS control group (Figure 6G). The expression of VEGF protein was detected *via* ELISA revealed a trend consistent with the mRNA results (Figure 6H). Vascular endothelial cells labeled with anti-CD31 antibody revealed that VEGF downregulation greatly reduced neovascularization (Figure 6I). The tumor infiltration of CD8^+^ T cells and CD4^+^ T cells was observed using immunofluorescence, which demonstrated that the combination treatment promoted the maximum tumor infiltration of CD8^+^ T cells and CD4^+^ T cells in tumors (Figure 6I), which was consistent with previous flow cytometry results (Figure 6C).

The activation of the immune memory effect can prevent tumor metastasis and recurrence (Liu et al., 2022). Therefore, T_EM_ cell level in spleens was measured after treatment. The level of T_EM_ cells was modestly increased in the HPVC group compared with that in the PBS group; however, the immune memory effect was significantly amplified when anti-PD-L1 therapy was introduced (Figure 6J). Encouraged by these positive results, the long-term immune memory effect was evaluated by inoculating treated mice on day 19 in the combination group and Naïve groups with 1 × 10^6^ fresh CT26 cells subcutaneously on the opposite back of the mice. The results revealed that tumor recurrence (2/5) was well controlled in the treated mouse group. Meanwhile, tumor growth in the recurrent mice was also effectively controlled (Figure 6K), suggesting the successful establishment of the immune memory effect after combination therapy.

### 3.7 Biocompatibility evaluation

**FIGURE 7:**
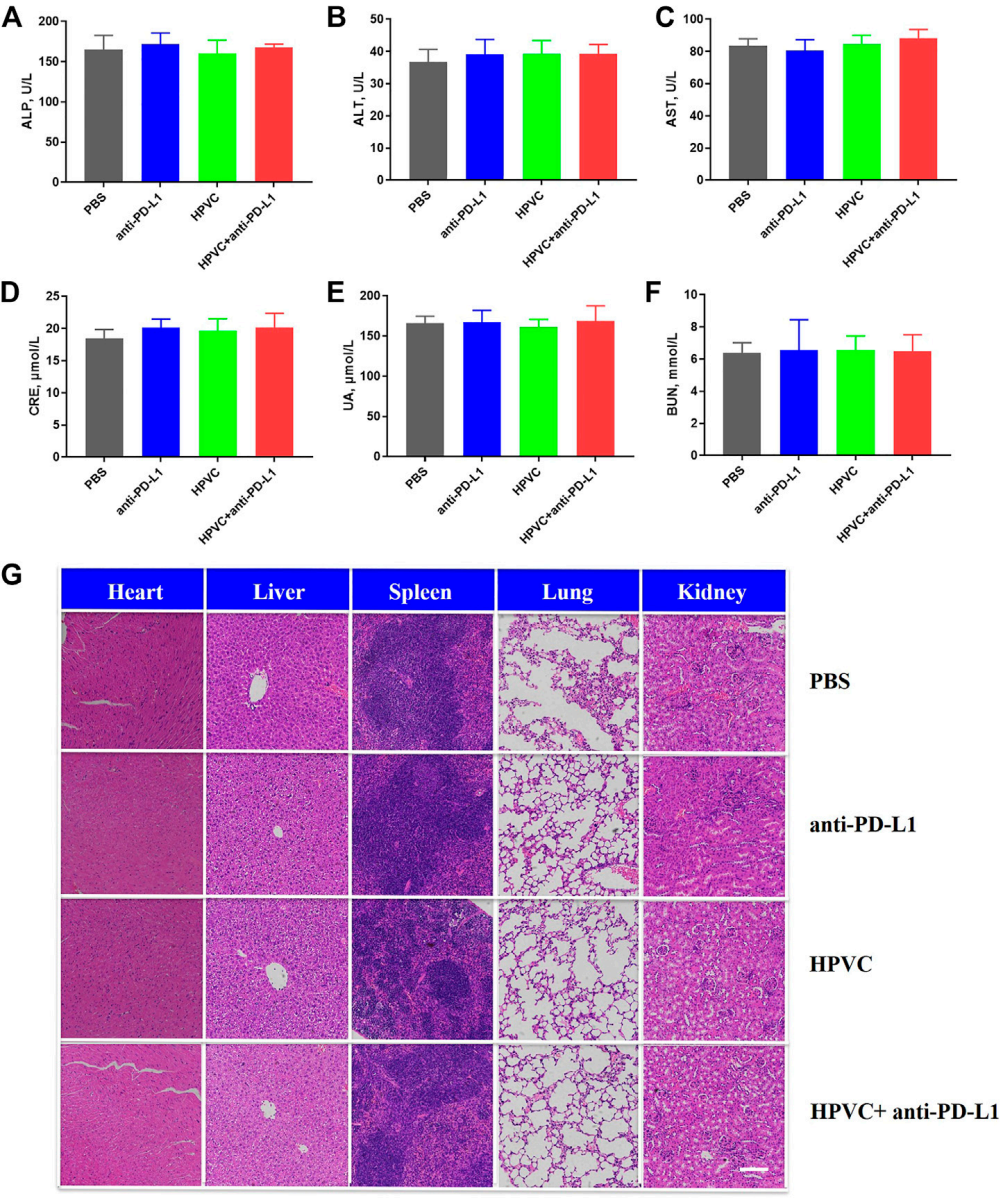
Biocompatibility evaluation. Hepatic function indexes **(A)** ALP, **(B)** ALT and **(C)** AST. Renal function indexes **(D)** CRE, **(E)** UA and **(F)** BUN. **(G)** H&E histological analysis of the main organs in different treatment groups on day 19. Scale bar = 200 μm.

Biocompatibility and effectiveness are the main two critical factors for clinical treatment. The effectiveness of the combination therapy strategy has been demonstrated previously. In the present study, the safety of the combination therapy was analyzed *via* various perspectives. First, there were no obvious differences in the body weights of mice of any treatment groups (Supplementary Figure S10). Next, the liver and renal function indices detected in the serum after treatment revealed no significant differences among all the groups, indicating the safety of the antitumor treatment strategy (Figures 7A–F). In addition, H&E staining was utilized to verify the histological morphology changes in the main organs showed no obvious mutative morphology (Figure 7G). All these results demonstrated that the combined treatment strategy was effective and has no significant side effects.

## 4 Conclusion

In summary, a synergistic strategy was established by combining *in situ* tumor vaccines, gene-mediated antiangiogenic therapy and anti-PD-L1 therapy. *In situ* tumor vaccines and antiangiogenic effects were achieved by codelivering shVEGF and CpG agonists *via* a delivery system comprising PEI25k and HA. HA/PEI/shVEGF/CpG nanoparticles effectively knocked down VEGF protein and stimulated antigen processing in cells *in vitro*. Furthermore, this strategy inhibited endothelial cell proliferation and angiogenesis, facilitated the infiltration of immune cells, and activated host immune responses. However, this strategy upregulated PD-L1 expression in tumor cells and aggravated the immunologic tolerance of tumor cells. To improve the immunosuppressive tumor microenvironment, therefore, an anti-PD-L1 monoclonal antibody was used to block the PD-1/PD-L1 immune checkpoint and reactivate the antitumor killing ability of infiltrated cytotoxic T cells. This combination therapy inhibited tumor growth and reduced tumor metastasis and recurrence, with no obvious side effects. This novel therapy is anticipated to provide a promising potential clinical therapeutic strategy for clinical antitumor treatment.

## Data Availability

The original contributions presented in the study are included in the article/Supplementary Material, further inquiries can be directed to the corresponding author.
