# Supplementary material for: Combination of immune checkpoint blockade and targeted gene regulation of angiogenesis for facilitating antitumor immunotherapy
Source: Front Bioeng Biotechnol. 2023 Mar 13;11:1065773. doi: 10.3389/fbioe.2023.1065773 (PMC10040836; doi:10.3389/fbioe.2023.1065773)
Supplement: Supplementary file 1 [file Table1.docx]

Supplementary Material


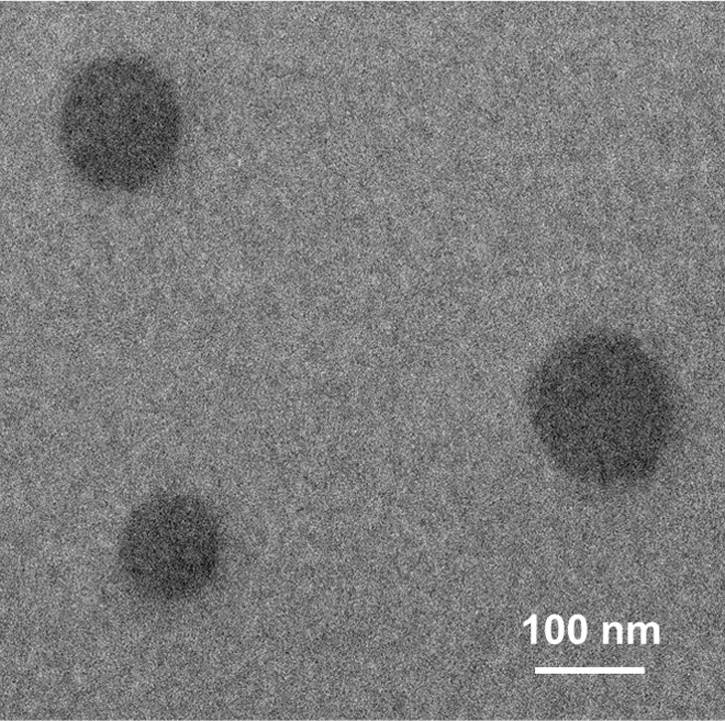


**Supplementary Figure 1.** TEM figure of HA/PEI25k/pGL3/CpG complexes at their weight ratio of 0.5:2:1:0.5. Scale bar = 100 nm.


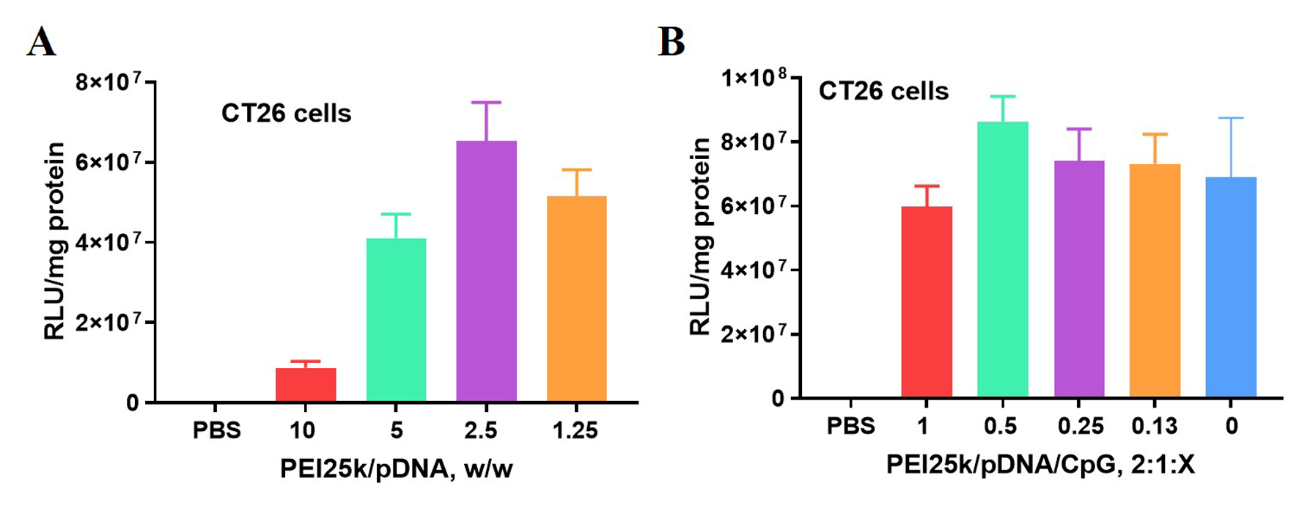


Supplementary Figure 2. Optimization of transfection conditions by luciferase reporter gene. (A) Gene transfection of PEI25k/pGL3 complexes at their different mass ratios. (B) Gene transfection of PEI25k/pGL3/CpG complexes in different amounts of CpG agonist.


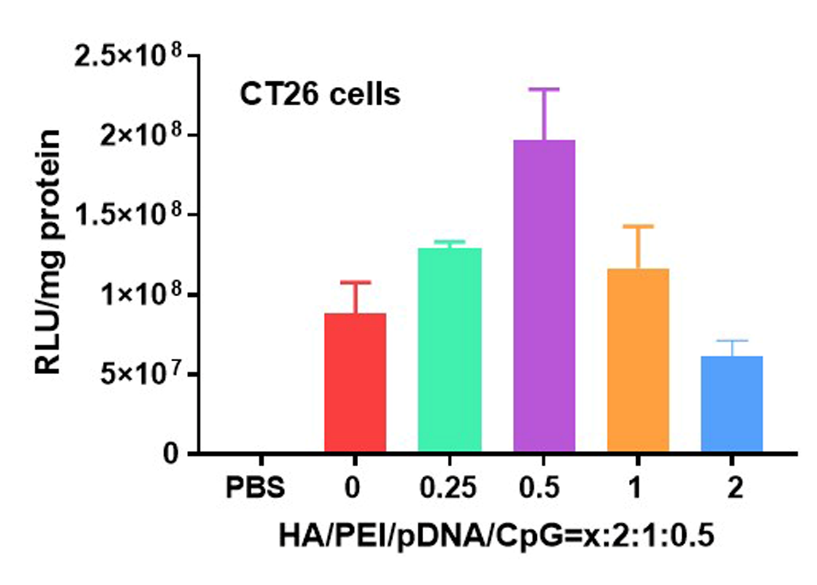


**Supplementary Figure 3.** Gene transfection efficiency of HA/PEI/pDNA/CpG nanoparticles at various contents of HA.


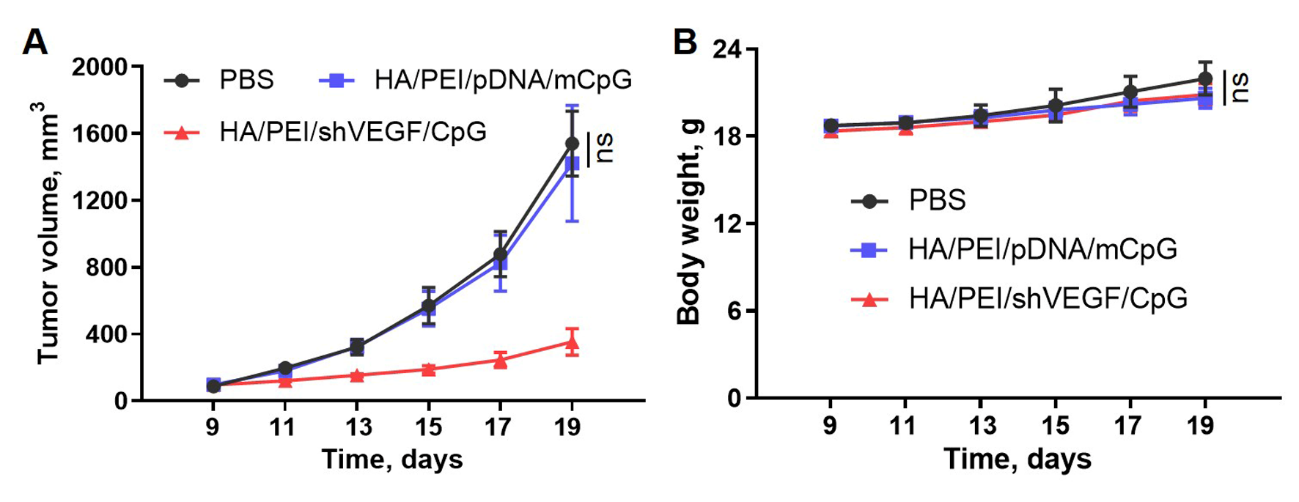


**Supplementary Figure 4.** Antitumor therapy by HA/PEI/pDNA/mCpG and HA/PEI/shVEGF/CpG nanoparticles. (A) Average tumor growth curves of mice in different treatment groups (n = 4). (B) Body weight curves of mice in different treatment groups (n = 4). pDNA: non-therapeutic pDNA; mCpG: CpG mimics with disrupted nucleic acid sequence.


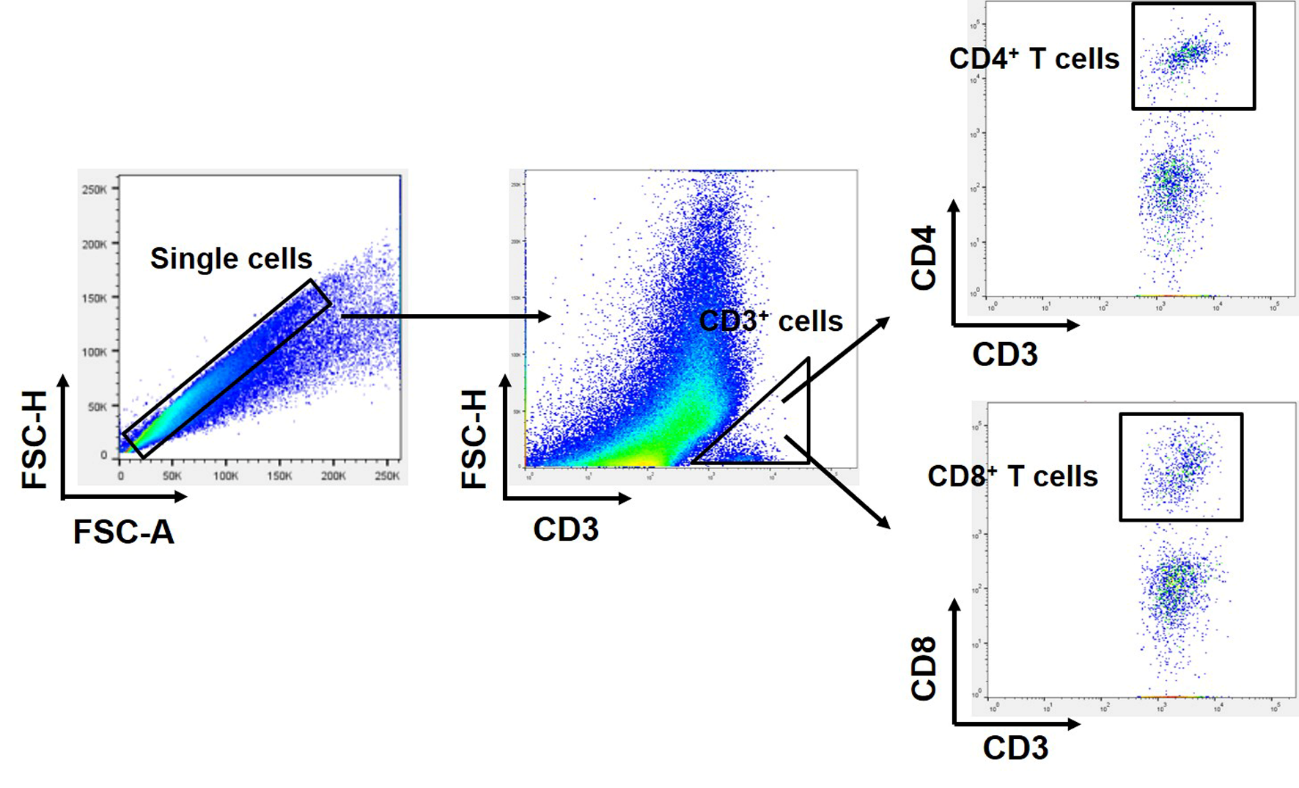


**Supplementary Figure 5.** The gating strategy for CD3+CD4+ and CD3+CD8+ T cells analysis in the tumors.

**
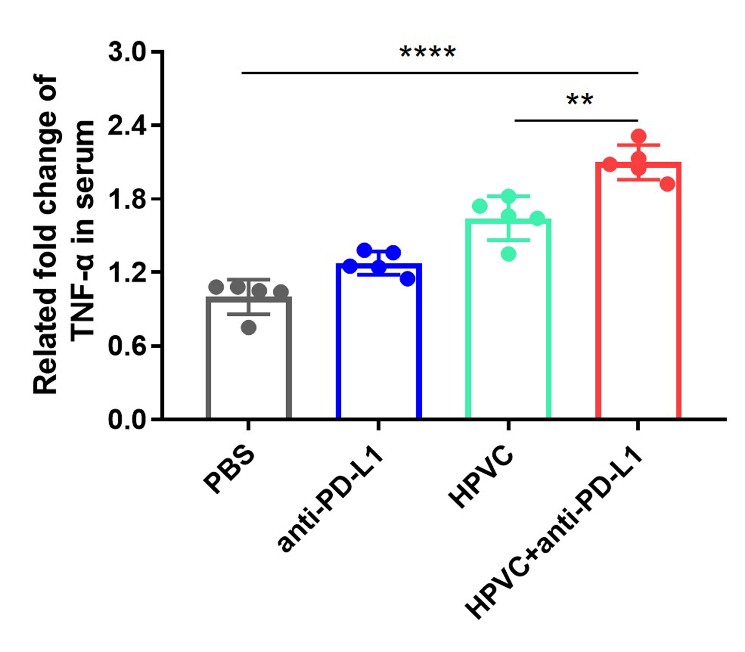
**

**Supplementary Figure 6.** Secretion of TNF-α in serum by ELISA. **P<0.01, ****P<0.0001.


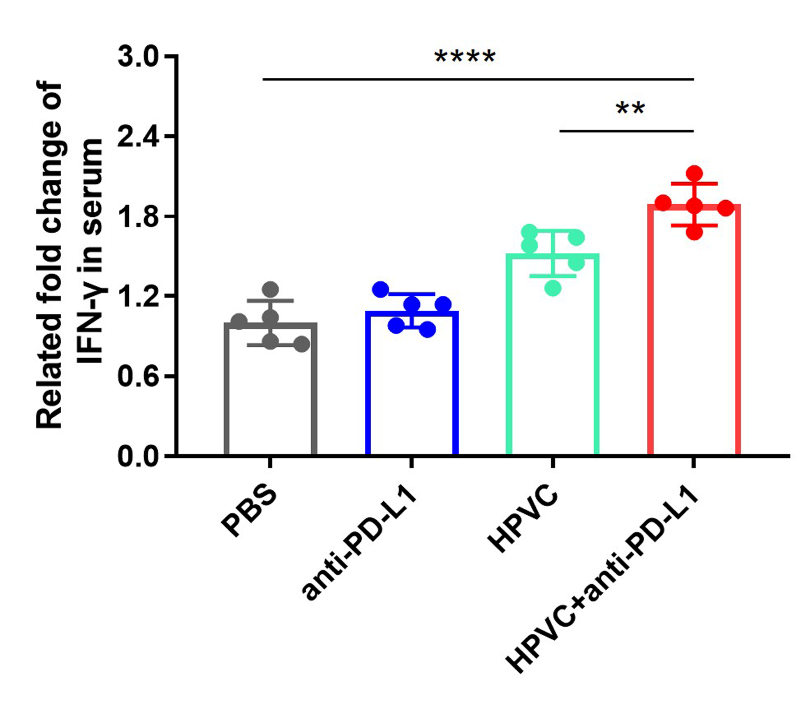


**Supplementary Figure 7.** Secretion of IFN-γ in serum by ELISA. **P<0.01, ****P<0.0001.


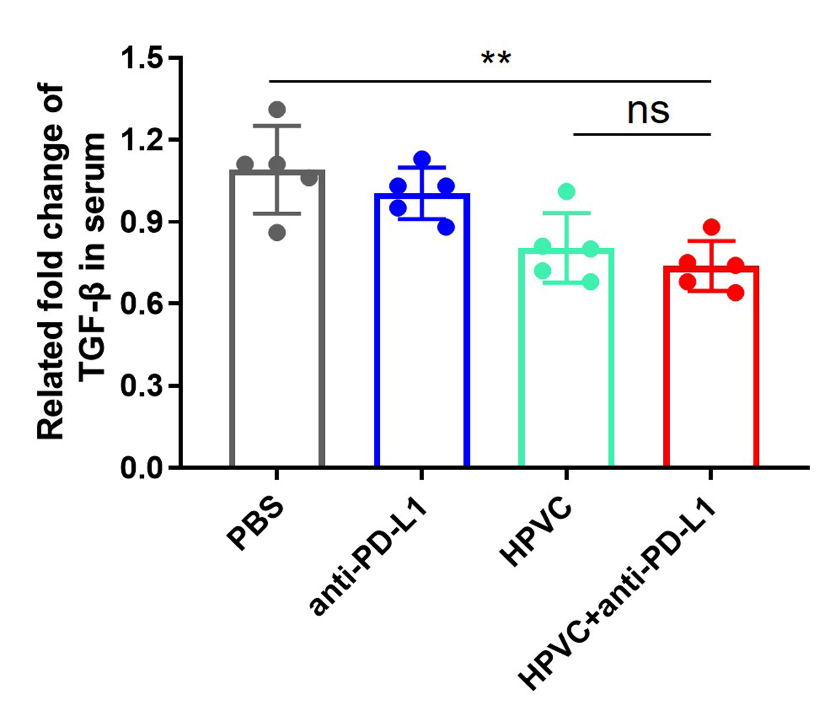


**Supplementary Figure 8.** Secretion of TGF-β in serum by ELISA. ns, no significant difference, **P<0.01.


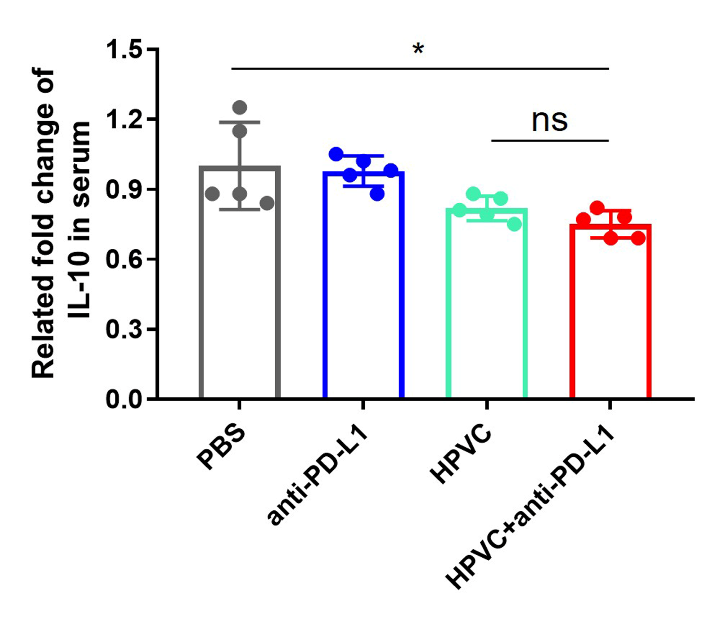


**Supplementary Figure 9.** Secretion of IL-10 in serum by ELISA. ns, no significant difference, *P<0.05.


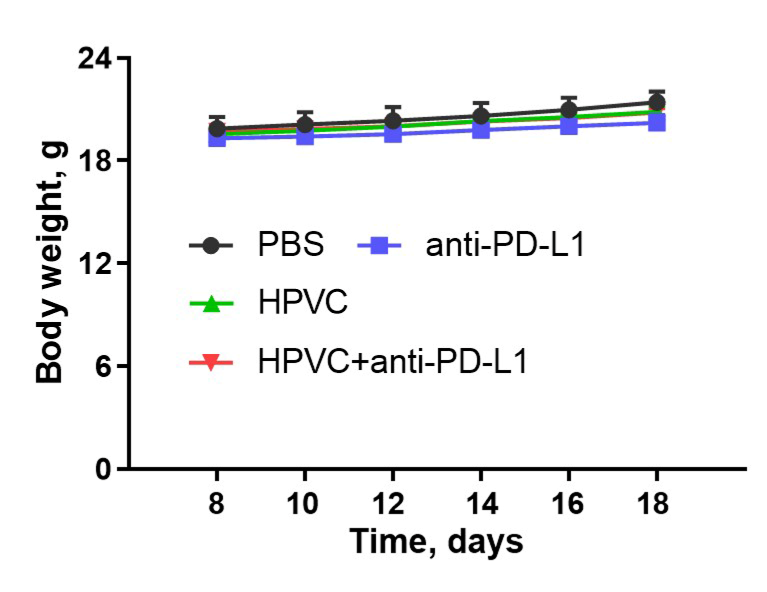


**Supplementary Figure 10.** Average body weight curves of all the treated groups.
